# Reiterating the Emergence of Noncoding RNAs as Regulators of the Critical Hallmarks of Gall Bladder Cancer

**DOI:** 10.3390/biom11121847

**Published:** 2021-12-08

**Authors:** Varsha Rana, Dey Parama, Elina Khatoon, Sosmitha Girisa, Gautam Sethi, Ajaikumar B. Kunnumakkara

**Affiliations:** 1 Cancer Biology Laboratory & DBT-AIST International Laboratory for Advanced Biomedicine (DAILAB), Department of Biosciences and Bioengineering, Indian Institute of Technology Guwahati, Assam 781039, India; ranavarsha21@gmail.com (V.R.); deyparama@iitg.ac.in (D.P.); elina@rnd.iitg.ac.in (E.K.); sosmi176106101@iitg.ac.in (S.G.); 2 Department of Pharmacology, Yong Loo Lin School of Medicine, National University of Singapore, Singapore 117600, Singapore

**Keywords:** GBC, ncRNAs, circRNAs, lncRNAs, miRNAs, snoRNA, signaling pathways, apoptosis, EMT, chemoresistance

## Abstract

Gall bladder cancer (GBC) is a rare and one of the most aggressive types of malignancies, often associated with a poor prognosis and survival. It is a highly metastatic cancer and is often not diagnosed at the initial stages, which contributes to a poor survival rate of patients. The poor diagnosis and chemoresistance associated with the disease limit the scope of the currently available surgical and nonsurgical treatment modalities. Thus, there is a need to explore novel therapeutic targets and biomarkers that will help relieve the severity of the disease and lead to advanced therapeutic strategies. Accumulating evidence has correlated the atypical expression of various noncoding RNAs (ncRNAs), including circular RNAs (circRNAs), long noncoding RNAs (lncRNAs), microRNAs (miRNAs), and small nucleolar RNAs (snoRNA) with the increased cell proliferation, epithelial–mesenchymal transition (EMT), invasion, migration, metastasis, chemoresistance, and decreased apoptosis in GBC. Numerous reports have indicated that the dysregulated expression of ncRNAs is associated with poor prognosis and lower disease-free and overall survival in GBC patients. These reports suggest that ncRNAs might be considered novel diagnostic and prognostic markers for the management of GBC. The present review recapitulates the association of various ncRNAs in the initiation and progression of GBC and the development of novel therapeutic strategies by exploring their functional and regulatory role.

## 1. Introduction

Despite the recent advancement in cancer research, cancer is among the most commonly occurring diseases and one of the primary causes of death globally [1,2,3,4,5]. GBC is the most common and aggressive form of biliary cancer. GBC spreads initially by lymphatic metastasis and directly invades the liver. During the initial stages, it is often associated with nonspecific symptoms and, thus, generally diagnosed when the disease has already progressed [6,7,8,9]. Recent studies have shown that GBC is multifactorial and closely associated with several risk factors, including chronic gallbladder infection, prolonged exposure to chemicals and heavy metals, and an unhealthy diet. Moreover, bile reflux, Mirizzi’s syndrome, and porcelain gallbladder play critical roles as predisposing factors for GBC. Although some studies have suggested that gallstones are also a crucial risk factor for GBC, their role in the development of this disease has not been fully determined [10,11]. Increasing evidence suggests that the lack of specific biomarkers for GBC diagnosis contributes significantly to the poor prognosis and poor survival of GBC patients [7]. Although surgery may well be curative at early stages, this cancer is generally diagnosed towards the advanced stage. Both surgical and nonsurgical modes of treatment remain primarily unsuccessful with the advancement of the disease [6,7,9]. Therefore, the exploration and identification of novel biomarkers for the management of GBC are urgently needed.

Regulatory ncRNAs are a cluster of RNAs that do not encode functional proteins. Initially, these ncRNAs were thought to control gene expression only at the post-transcriptional level [12,13,14,15]. However, studies have revealed that they involve a network of internal signals that control the expression of numerous genes regulating the physiological and developmental processes. Evidence suggests that these regulatory ncRNAs also play a vital role in epigenetic control [14,15]. Additionally, studies have evidenced the pivotal role of ncRNAs in cancer. Their aberrant expression can contribute to tumor growth and metastasis [16]. Hence, in the current review, we aim to discuss the emerging role of ncRNAs in GBC to explore its potential as an effective diagnostic and therapeutic target for managing this disease.

## 2. Noncoding RNAs (ncRNAs) in GBC

**Figure 1:**
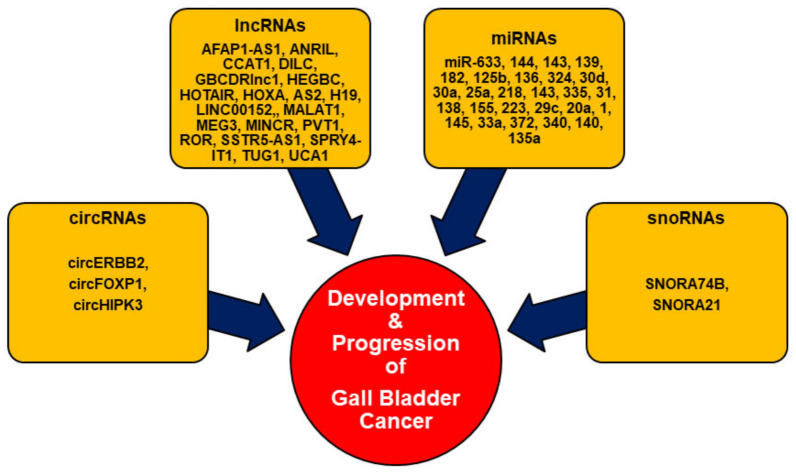
Regulatory ncRNAs in GBC.

Noncoding RNAs (ncRNAs) are broadly classified into housekeeping and regulatory ncRNAs. Regulatory ncRNAs consist of small ncRNAs (sncRNAs) (<200 nucleotides in length) and long lncRNAs (>200 nucleotides in length). The sncRNAs mainly comprise miRNA, small interfering RNAs (siRNAs), and piwi-interacting RNAs (piRNAs). Nevertheless, some ncRNAs, such as promoter-associated transcripts (PATs), enhancer RNAs (eRNAs), and circRNAs, vary in length and thus can simultaneously be classified under sncRNAs and lncRNA [17,18,19,20]. Several studies have demonstrated the close association of different regulatory ncRNAs such as circRNAs, lncRNAs, miRNAs, and snoRNAs in the initiation and progression of GBC (Figure 1) [21,22,23,24].

### 2.1. Circular RNAs (circRNAs)

Circular RNAs (circRNAs) are a class of lncRNAs having covalent bound closed-loop structures formed as a result of the back-splicing of premessenger RNAs (pre-mRNAs) [22,25]. Although the function of all the existing circRNAs is not entirely clear, several circRNAs act as “microRNA sponge” and “RNA binding protein sponge”. The aberrant expressions of numerous circRNAs, which can act as a tumor suppressor or oncogene, are often reported in different types of cancer, such as breast cancer, cervical cancer, gastric cancer, and prostate cancer. To date, only a few circRNAs have been found to regulate the progression of GBC. Moreover, they might be associated with the sponging of miRNAs or have a different regulatory axis for the development and progression of GBC [22,25,26]. The upregulation of some circRNAs is associated with the poor prognosis and poor survival of the GBC patients [27,28]. For instance, one study has reported the positive correlation between the augmented expression of circular–mitochondrial translation optimization 1 (circ-MTO1) in the plasma of GBC patients and poor progression-free and overall survival [27]. Further, another circRNA, circFOXP1, was reported to be upregulated in GBC and was found to regulate the expression of pyruvate kinase, liver, and RBC (PKLR) by sponging miR-370. The upregulation of this circRNA was also shown to induce the Warburg effect in GBC cell lines [28].

### 2.2. Long Noncoding RNAs (lncRNAs)

Long noncoding RNAs (lncRNAs) are a class of ncRNA 200 nucleotides or greater in length and are relatively stable compared with circulating tumor DNA (ctDNA) due to being predominantly secreted in vesicles or forming complexes with other proteins. Even though the lncRNAs do not encode for proteins or peptides, they play varied roles in modulating the expression and function of various genes at transcriptional, translational, and post-translational levels [29,30,31,32,33]. Numerous studies have evidenced the pivotal role of lncRNAs in several cellular processes, including cell differentiation, organogenesis, and tissue homeostasis. Therefore, lncRNAs are closely associated with cancer and cardiovascular diseases [33,34]. Multiple strands of evidence suggested that lncRNAs regulate various transcription factors and chromatin–remodeling complexes. Studies have shown that lncRNAs interact directly with the promoter regions and function as miRNA sponges [33,34]. Further, the upregulation of several lncRNAs, such as LINC00152, metastasis-associated lung adenocarcinoma transcript 1 (MALAT1), plasmacytoma variant translocation 1 (PVT1), urothelial carcinoma-associated 1 (UCA1), etc., have been reported in GBC. Beyond GBC, several lncRNAs, and in particular, the levels of UCA1 and MALAT-1, have been found significantly higher in other cancers such as melanoma compared to controls and were correlated to the stage of the disease. The aberrant expressions of several lncRNAs are correlated with poor prognosis, tumor progression, and lymph node invasion in GBC [23,30,35,36,37].

### 2.3. MicroRNAs (miRNAs)

MicroRNAs (miRNAs) are small endogenous ncRNAs having a length of approximately 20–25 nucleotides [38,39]. These ncRNAs are known to regulate the expression of several genes post-transcriptionally by binding directly to the 3′ untranslated regions (3′UTRs) of the corresponding mRNAs. This process leads to the induction of mRNA degradation and suppression of protein translation. miRNAs can function as both tumor promoters and suppressors [39,40] and were found to regulate crucial pathways and genes related to tumorigenesis and metastasis. Dysregulation of miRNA expression is commonly involved in different cancers, including breast cancer, colorectal cancer, prostate cancer, and others [41,42,43,44,45]. For instance, several studies highlight the potential utility of miRNAs as biomarkers in either tissues or blood for assessing response to the agents implemented in colorectal cancer, including the 5-fluorouracil based therapies and EGFR inhibitors [46]. In GBC, the miRNAs, such as miR-182 and miR-155, were reported to be upregulated, which positively correlated with lymph node metastasis and poor prognosis in the patients [24,47]. A similar positive correlation was also observed in GBC patients with diminished expression of specific miRNAs such as miR-125b, miR-136, miR-30a-5p, etc. [48,49,50].

### 2.4. Small Nucleolar RNAs (snoRNAs)

Small nucleolar RNAs (snoRNAs) are a class of regulatory ncRNAs ranging from 60 to 300 nucleotides in length and, therefore, can be classified under both sncRNAs and lncRNA. snoRNAs are primarily located in the nucleolus. Recent studies have reported the role of snoRNAs in regulating GBC both in vitro and in vivo. Additionally, several studies have reported their role as oncogenes and tumor suppressors in GBC [21,51].

## 3. Deregulation of ncRNAs in Different Signaling Pathways

**Figure 2:**
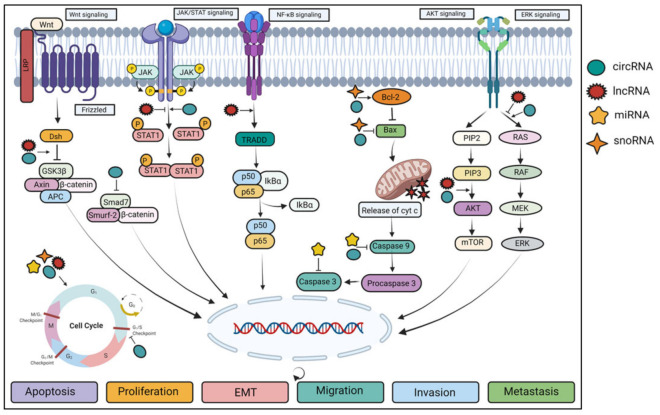
Role of ncRNAs in regulating different signaling pathways in GBC.

The ncRNAs have emerged as important regulators of critical signaling pathways and prognostic markers in several cancers, including GBC (Figure 2). Accumulating evidence has demonstrated that lncRNAs play a significant regulatory role in GBC tumorigenesis (Table 1) [21,22,23,24,26,28,36,37,47,48,50,51,52,53,54,55,56,57,58,59,60,61,62,63,64,65,66,67,68,69,70,71,72,73,74,75,76,77,78,79,80,81,82,83,84,85,86,87,88,89,90,91,92,93,94]. For instance, Cai et al. reported that upregulated expression of lncRNA ENST00000425894 termed gallbladder cancer drug resistance-associated lncRNA1 (GBCDRlnc1) activated autophagy and doxorubicin-resistance in GBC cells. They further demonstrated that interaction of GBCDRlnc1 with phosphoglycerate kinase 1 (PGK1) prevented PGK1 ubiquitination, which eventually led to the elevated expression of autophagy-related genes ATG5-ATG12 [56]. Another study reported the positive association of lncRNA PVT1 with malignancies and poor overall survival in GBC. Further, PVT1 was reported to act as a competing endogenous RNA (ceRNA) that sponged miR-143, thereby modulating the expression of hexokinase 2 (HK2) [35]. Similarly, lncRNA downregulated in liver cancer stem cells (lnc-DILC) was found to drive GBC stem cells expansion through the Wnt/β-catenin pathway [55]. Wang et al. revealed the involvement of MYC-induced lncRNA (MINCR) in promoting the expression of enhancer of zeste homolog 2 (EZH2) via targeting endogenous miR-26a-5p in GBC [66]. Furthermore, binding of deleted in malignant brain tumors 1 (DMBT1), a tumor-suppressor gene, with lncRNA colorectal neoplasia differentially expressed (CRNDE), and cellular inhibitor of apoptosis protein 1 (c-IAP1) was found to promote PI3K-AKT signaling pathway in GBC [95]. In addition, transcription factor specificity protein 1 (SP1)-mediated upregulation of lncRNA LINC00152 via PI3K/AKT pathway was reported to be associated with tumor cell growth and metastasis in GBC [61]. Studies have reported the oncogenic role of lncRNA high expressed in gallbladder cancer (HEGBC) and MALAT1 in GBC through modulation of IL-11/STAT3 and ERK/MAPK signaling pathway, respectively [23,57]. Furthermore, MALAT1 acted as a ceRNA by negatively regulating the expression of MCL-1 via sponging miR-363-3p in GBC [64]. Additionally, lncRNA SNHG6 was found to modulate the Hedgehog signaling pathway via targeting miR-26b-5p, thus affecting proliferation, invasion, and EMT in GBC cells [96]. Moreover, Bao et al. suggested that the lncRNA maternally expressed gene 3 (MEG3) regulated proliferation and apoptosis of GBC cells via induction of NF-κB signaling [97].

In addition to lncRNA, numerous studies have reported the involvement of the ectopic expression of miRNAs in GBC development through the modulation of various signaling pathways. For example, a study reported that miR-20a triggered the EMT and metastasis in GBC cells via targeting the Smad7/β-catenin axis [87]. Further, Goeppert et al. suggested the functional role of miR-145-5p in activating signal transducer and activator of transcription 1 (STAT1) signaling in biliary tract cancer [98]. In another study, miR-663a was reported to attenuate epithelial membrane protein-3 (EMP3) expression and activate MAPK/ERK pathway, thereby mediating the development of GBC [72]. Ishigami et al. demonstrated the involvement of the IL-6/STAT-3 signaling pathway in the growth of bile duct cancer cells and miR-31 expression [99]. Moreover, overexpression of miR-31 enhanced cisplatin chemosensitivity of GBC cells via the Src/Akt/Bax/Bcl-2 signaling pathway [83]. Another study reported that overexpression of miR-136 and miR-33b promoted apoptosis and inhibited angiogenesis and EMT in GBC cells via suppressing mitogen-activated protein kinase kinase 4 (MAP2K4)-dependent Jun NH2-terminal kinase (JNK) signaling pathway and ciliary rootlet coiled-coil protein (CROCC), respectively [48,100]. Furthermore, miR-143-5p and miR-29c-5p impeded EMT and exerted apoptotic effect in GBC cells via blocking the expression of HIF-1α and MAPK/ERK pathway, respectively [75,86]. Another miRNA, miR-33a, displayed its tumor-suppressive activity by suppressing IL-6-mediated tumor progression via binding Twist in GBC [89]. Similarly, miR-372, miR-135a, and miR-101 were reported to regulate GBC progression via targeting chloride intracellular channel 1 (CLIC1), very low-density lipoprotein receptor (VLDLR)–p38 axis, zinc finger protein X-linked (ZFX), MAPK/ERK, and Smad pathways [91,94,101]. Moreover, miR-140-5p negatively regulated the oncogenic function of Septin 2, a tumor-promoting gene, in biliary tract cancers [93].

Moreover, a snoRNA, SNORA74B, was reported to exert oncogenic activity in GBC through suppressing pleckstrin homology domain leucine-rich repeat protein phosphatase (PHLPP) (an endogenous inhibitor of Akt) and activating Akt/mTOR signaling pathway [51]. These studies suggest the significant role of ncRNAs as a therapeutic strategy for the treatment of GBC.

## 4. Effect of ncRNAs in Different Hallmarks of Cancer

**Figure 3:**
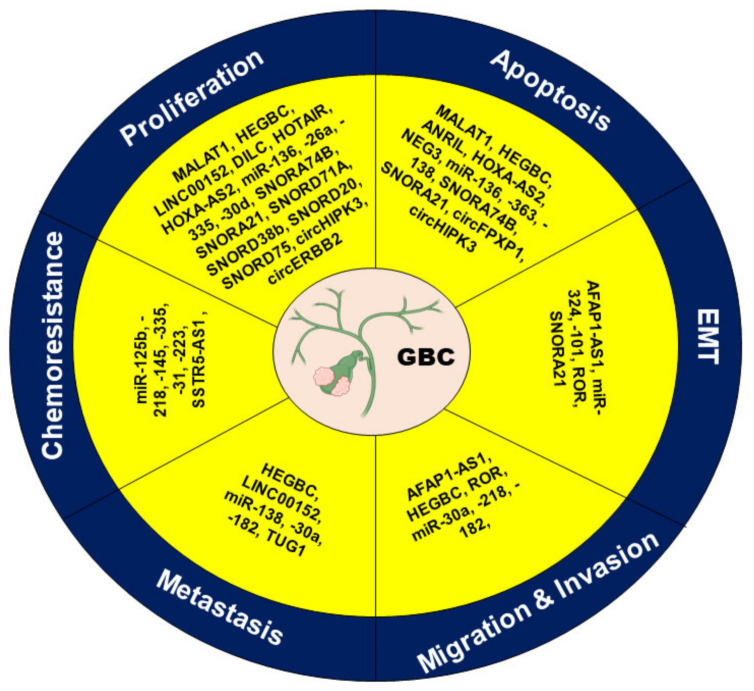
Different ncRNAs modulating the critical hallmarks in GBC.

The ncRNAs are shown to regulate the critical hallmarks of cancer. The role of these ncRNAs involved in modulating the processes leading to the development and progression of GBC is described in Figure 3.

### 4.1. Apoptosis

Apoptosis, also regarded as programmed cell death, is a crucial process for controlling cancer progression, and cancer cells are associated with a decreased rate of apoptosis compared with normal cells [102,103,104,105,106]. Apoptotic cell death is associated with the induction of caspases, the release of cytochrome c (cyt C), and proapoptotic proteins [107,108,109]. Numerous studies have reported the involvement of ncRNAs in the regulation of apoptosis in GBC. For instance, the circRNA circHIPK3 was reported to be upregulated in GBC, and it was shown to act via sponging the miR-124 that depleted its expression [22]. Further, another circRNA circFOXP1 was found to show similar activity in GBC via sponging miR-370, and the abnormal expression of the circRNA was also found to induce the Warburg effect in GBC cells [28].

Additionally, a study by Liu B et al. reported that the expression of the lncRNAs antisense ncRNA in the INK4 locus (ANRIL) and MEG3 was upregulated and downregulated, respectively, in GBC cells, and silencing of ANRIL and overexpression of MEG3 promoted apoptosis in GBC cells [53]. Similarly, silencing the lncRNA, HOXA-AS2 also significantly suppressed the proliferation of GBC cells by inducing G1 phase arrest and apoptosis [59]. Further, overexpression of HEGBC suppressed apoptosis in SGC-996 and NOZ cells [57].

Like lncRNA, miRNAs are also reported to modulate the factors that regulate apoptosis in GBC. For instance, the elevated expression of miR-136 induced apoptosis in in vivo model of GBC via the downregulation of MAP2K4 [48]. Further, depletion of MCL-1, a target of miR-363-3p, promoted apoptosis and substantially reduced the cell proliferation in NOZ cells [64]. Another study evaluating the tumor-suppressive role of miR-138 showed that it targeted the 3′UTR of Bag1, an antiapoptotic protein, and inhibited its expression. Moreover, overexpression of miR-138 led to miR-138-mediated Bag1 inhibition and regulated the expression of apoptosis-associated proteins such as Bcl-2 and Bax [84].

Similarly, researchers have investigated the role of snoRNA in the regulation of apoptosis and reported that a snoRNA, SNORA74B enhanced apoptosis in preclinical models of GBC by upregulating the expression of Bax, cleaved caspase-3, and cytosolic C (cyt C), and downregulating the expression of Bcl-2 and mitochondrial cyt C [51]. Further, overexpression of another snoRNA, SNORA21, in GBC-SD and G415 cells also resulted in a similar effect on apoptosis-associated proteins Bax, cleaved caspase 3, and Bcl-2 [21].

### 4.2. Cell Proliferation

Cell proliferation is another critical process disrupted in cancer cells leading to atypical proliferative ability [110,111,112,113,114]. Research has shown the role of ncRNAs, including several circRNAs, in the survival and proliferation of cancer cells. One study has reported that silencing FOXP1 inhibited the proliferation of GBC cells and induced G1-S phase arrest [28]. Further, circHIPK3 was reported to impart its activity by sponging the tumor suppressor miR-124, and knockdown of cirHIPK3 elevated the expression of miR-124. Moreover, the knockdown also induced a decrease in the expression of miR-124 targeted cyclin-dependent kinase 6 (CDK6) and rho-associated protein kinase 1(ROCK1) [22]. However, a few circRNAs might also act beyond the miRNA sponge. Thus, circERBB2 promoted GBC proliferation by increasing the activity of polymerase-I and transcription of the active ribosomal DNA (rDNA) via the regulation of proliferation-associated 2G4 (PA2G4) [26].

The lncRNAs are also reported to regulate the proliferation and survival of GBC. The overexpression of MALAT1 was often associated with a poor prognosis of patients. Recent studies have also shown that upregulated expression of MALAT1 enhanced cell proliferation and metastasis in GBC cells, and silencing of the same reduced the proliferative factors, Ki67 and proliferating cell nuclear antigen (PCNA). Additionally, the study also showed that MALAT1 inhibited the expression of the tumor suppressor ABI3BP in GBC cells [63]. Although the mechanism of action of MALAT1 is unknown, studies have shown that the lncRNA might induce cell proliferation in GBC cells via activation of the ERK/MAPK pathway. This is evidenced by the knockdown of MALAT1, which significantly suppressed the aforementioned pathway by inactivation of ERK 1/2, MEK1/2, MAPK, and JNLK 1/2/3 [23]. Another lncRNA, AFAP1-AS1, was overexpressed in GBC that significantly correlated with the poor prognosis of the GBC patients, and the silencing of AFAP1-AS1 resulted in the decrease of GBC cell proliferation [52]. Additionally, the knockdown of lncRNA HEGBC drastically suppressed the proliferation of GBC-SD and EH-GB2 cells [57]. Furthermore, a study reported that specificity protein 1 (SP1)-mediated upregulation of the lncRNA LINC00152 in GBC in vitro and in vivo induced proliferation and tumor growth via the PI3K/AKT pathway [61]. Moreover, another lncRNA DILC induced progression of GBC cells via Wnt/β-catenin activation [55]. Several lines of evidence further suggested that the knockdown of various other lncRNAs such as HOTAIR, HOXA-AS2, and ROR are also involved in regulating the proliferation of GBC cells [58,59,68].

Studies exploring the role of miRNAs in regulating cell survival and proliferation have suggested that the miRNA, miR-136, expression was downregulated in GBC, and an increase in their expression suppressed cell proliferation [48]. A study further demonstrated that a depleted expression of miR-26a has been observed in GBC, and it is positively correlated with neoplasm histological grade. Furthermore, miR-26a led to the inhibition of proliferation of GBC cells by binding to its downstream target high mobility group AT-hook 2 (HMGA2). Moreover, miR-26a also abrogated G1/S transition in GBC cells [79]. Another study showed that miR-335 was significantly downregulated in GBC cells, and the introduction of miR-335 mimics the expression of cell cycle-associated proteins, such as cdc2 and cdc25. Furthermore, an increased miR-335 expression also suppressed cell viability and induced cell cycle arrest in GBC cells. Growing evidence suggested that miR-335 inhibited cancer cell viability and induced cell cycle arrest by inhibiting the expression of myocyte enhancer factor 2D (MEF2D) [82]. Further, the depleted expression of miR-30d-5p is associated with a poor prognosis and survival of GC patients. It inhibited GBC progression by negatively regulating the expression of lactate dehydrogenase-A (LDHA) [78].

Recent studies have also highlighted the role of snoRNAs in GBC tumorigenesis. A study reported that snoRNAs such as SNORA74B, SNORA21, SNORD71A, SNORD38b, SNORD20, and SNORD75 were significantly upregulated in GBC tissue. Further, the knockdown of the SNORA74B inhibited proliferation and induced G1 phase arrest in GBC cells by regulating the expression of G1 proteins such as p21, p27, and cyclin D1 [51]. Contrary to SNORA74B, SNORA21 acted as a tumor suppressor, and its expression was downregulated in GBC. Moreover, the upregulation of SNORA21 suppressed proliferation and induced G1-G0 arrest of GBC-SD cells. Additionally, it decreased the expression of c-Myc and cyclin D1 [21].

### 4.3. EMT

EMT is a transcriptionally regulated process of transforming epithelial cells into mesenchymal cells by losing their ability to polarize and adhere and gaining the ability for invasion and migration [115,116]. It plays a crucial role in cancer initiation, invasion, and metastasis [117,118,119,120]. Recent studies have shown that the deregulation of several ncRNAs, such as lncRNAs and miRNAs, drives EMT in different cancers, including GBC [36,77,121,122]. Aligned with this, the knockdown of the lncRNA, AFAP1-AS1 was found to inhibit EMT progression by modulating the levels of Twist1, vimentin, and E-cadherin in GBC [52]. Further, the lncRNA ROR was reported to be upregulated in GBC and plays a vital role in regulating EMT progression. Silencing of the same regulated the levels of EMT markers such as E-cadherin, Twist1, and vimentin in SGC-996 and NOZ cells [68].

Additionally, studies have reported the involvement of miRNAs in the regulation of EMT in GBC. One such study reported that elevated expression of miR-324-5p in GBC plays a crucial role in EMT by elevating the levels of E-cadherin and suppressing the levels of N-cadherin and vimentin [77]. Further, the upregulation of the tumor suppressor miRNA, miR-101, in GBC cells significantly repressed the initiation of the MAPK/ERK and Smad pathways, which in turn resulted in the suppression of TGF-β-induced EMT [101]. Furthermore, the miRNA-200 family members are known to be involved in the regulation of EMT and might be involved in the mechanism of docetaxel resistance. Moreover, miRNA-1246 is a prostate cancer tumor suppressor miRNA that inhibits EMT, cellular proliferation, and survival and promotes apoptosis [123].

Similarly, snoRNAs are also reported to play a vital role in regulating EMT. For example, a study reported that overexpression of the tumor suppressor snoRNA, SNORA21 suppressed EMT by regulating the expression of the EMT regulatory proteins E-cadherin, N-cadherin, and vimentin [21].

### 4.4. Invasion and Migration

The invasion and migration processes are critical to facilitate the spread of a tumor to distant sites [124,125]. These critical processes were demonstrated to be regulated by numerous ncRNAs. One study reported that MALAT1 regulated the invasion and migration of GBC cells via activating the ERK/MAPK pathway [23]. Another study evaluating the regulatory role of MALAT1 in GBC models showed that the lncRNA downregulated the expression of ABI3BP, an important protein present in the extracellular matrix [63]. Further, knockdown of the lncRNAs AFAP1-AS1, HEGBC, and ROR also decreased invasion and migration in GBC cells [52,68]. Furthermore, it was demonstrated that the overexpression of the lncRNA, CCAT1 in GBC tissues induced miRNA-218-5p-mediated expression of Bmi1 and promoted proliferation and migration of cells [54].

In addition to the lncRNAs, the miRNAs are also known to control these critical processes in GBC. For instance, a study showed that miR-30a-5p mimics inhibited migration and invasion in GBC-SD and NOZ cells [49]. Likewise, exosomal miR-182 was also reported to induce migration and invasion of GBC cells by inhibiting the reversion-inducing-cysteine-rich protein with kazal motifs (RECK) protein, which is a regulator of extracellular matrix remodeling [47].

### 4.5. Metastasis

Metastasis is the critical process involved in spreading cancerous cells from one place to the neighboring tissues and distant organs [126]. The involvement of ncRNAs in the regulation of metastasis is reported by various studies. For example, a study reported that the depleted expression of HEGBC inhibited tumorigenesis and metastasis in in vivo models of GBC via the modulation of the IL-11/STAT3 signaling pathway [57]. Further, overexpression of LINC00152 significantly upregulated the invasion and migration of GBC-SD via the PI3K/AKT pathway, thereby promoting GBC cell metastasis [61]. Another study reported that LINC00152 acted as a ceRNA and got directly bound to miR-138. This binding enhanced the levels of HIF-1α and promoted metastasis and EMT progression in GBC [36]. Moreover, another lncRNA, TUG1, induced metastasis and EMT progression in GBC by sponging miR-300 [71].

In addition, studies evaluating the role of miRNA in GBC suggested that depleted expression of miR-30a-5p was inversely correlated with tumor size and lymph node metastasis, indicating that it might act as a tumor suppressor in GBC. It also inhibited metastasis in GBC by negatively regulating the expression of E2F7 [49]. Another study reported the elevated levels of miR-182 and exosomal miR-182 in GBC tissues and its correlation with tumor-node-metastasis (TNM) stages [47].

### 4.6. Chemoresistance

GBC is an aggressive malignant cancer resistant to a variety of chemotherapeutic drugs. The common chemotherapeutics administered to GBC patients include cisplatin, gemcitabine, oxaliplatin, and 5-fluorouracil [85,127,128,129]. However, it is well-known that in cancer cells’ chemoresistance to various chemotherapeutic agents greatly contributes to a decrease in sensitivity to chemotherapy [130,131,132,133,134]. Increasing evidence highlights the role of ncRNAs as regulators of chemoresistance. Several studies show that the deregulation of ncRNAs, particularly miRNAs, modulates the expression of drug resistance-related genes and regulates chemosensitivity or chemoresistance in GBC cells [76,80]. Additionally, miRNAs have been found to induce chemoresistance in other malignancies as well. For instance, 5-fluorouracil, leucovorin, and oxaliplatin (FOLFOX) resistance in advanced CRC is significantly associated with upregulation and downregulation of several serum miRNAs [46].

A study reported that the lncRNA SSTR5-AS1 is overexpressed in GBC cells and negatively correlated with the overall survival rate of patients. SSTR5-AS1 facilitated resistance to gemcitabine via the inhibition of apoptosis and regulated the protein levels of the non-POU domain-containing octamer-binding (NONO), essential for the SSTR5-AS1-mediated gemcitabine resistance [69].

Along with lncRNAs, miRNAs are also found to regulate chemoresistance in GBC. For instance, a study reported that miR-125b-5p chemosensitized GBC cells to cisplatin [76]. Another miRNA, miR-218-5p, acted as a tumor suppressor in GBC, and its low expression was negatively associated with a poor prognosis. It also played a critical role in chemosensitivity in GBC. Decreased expression of miR-218-5p confers chemoresistance against gemcitabine while its overexpression sensitizes GBC cells to gemcitabine. Moreover, this chemosensitizing potential of miR-218-5p is protein kinase C epsilon (PRKCE)-dependent. miR-218-5p induces gemcitabine sensitivity in GBC by abrogating PRKCE-induced increased levels of multidrug resistance 1/Permeability-glycoprotein (MDR1/P-gp) [80]. Another study reported that low expression of miR-145 induced MRP1-mediated chemoresistance to cisplatin in GBC cells. In contrast, a high expression of miR-145 sensitized GBC cells to cisplatin [81]. Further, downregulation of miR335 promoted resistance in GBC-SD and SGC-996 cells to 5-fluorouracil and overexpression of the same induced sensitivity against 5- fluorouracil by reducing the expression of the drug resistance-related proteins such as ATP-binding cassette transporter B1 (ABCB1) and ATP-binding cassette G2 (ABCG2) [82]. Another study reported that the expression of miR-31 was decreased in cisplatin-resistant GBC-SD and NOZ cells. It further stated that miR-31-mediated cisplatin resistance in GBC is Src-dependent. miR-31 expression is negatively correlated with Src, and the depletion of Src in cisplatin-resistant cells restored sensitivity [83]. Another miRNA, miR-223, sensitizes GBC cells to microtubule docetaxel, a microtubule targeting chemotherapeutic drug, and one study reported that miR-223-mediated chemosensitivity is disrupted in GBC cells due to the upregulation of STMN1 expression [85].

## 5. Challenges Associated with Clinical Applications of ncRNAs

Numerous studies in recent years have investigated the emerging critical role of ncRNAs in GBC and other diseases. Although ncRNAs have shown immense potential as a potential diagnostic marker and therapeutic target, there is still a need for extensive research and findings that can be translated into clinical application. There are several challenges associated with the development of miRNA-based clinical applications in cancer and other diseases. Some of the major challenges include delivery systems, administration routes, dosage concerns, as well as off-target effects. To overcome these difficulties, detailed pharmacokinetics studies should be conducted in in vivo models and validated in clinical studies. Optimizing the dosage for miRNA-based drugs is a challenge as an excessive dosage of miRNA or antimiRs might lead to an amplification of the off-target effects as well as nonspecific immunological responses. In addition, tissue-specific delivery is essential for adequate cellular uptake of synthetic oligonucleotides drugs. Although nanoparticle-based miRNA delivery has shown promising results in preclinical studies, in terms of enhanced therapeutic efficacy and reduced side effects, virtually no findings have been tested in clinical settings. Therefore, it is essential to test these miRNA-delivery systems in clinical studies to establish a functionally stable, safer, and biocompatible system. Another challenge is the administration routes of miRNA-based drugs. At present, only intravenous or subcutaneous administration of miRNA mimics or antimiRs can be performed. As the half-life of these oligonucleotide drugs is only a few minutes, the development of oral delivery vehicles is imperative [135,136,137,138].

Although a handful of preclinical studies have been conducted on the potential of ncRNAs in GBC, their utility in therapy is limited. Thus, extensive studies aiming to understand the acting mechanism of ncRNAs, pharmacokinetics dynamics, and optimal delivery systems are crucial to translate the findings in therapeutics and overcome the existing challenges associated with their clinical application.

## 6. Future Prospects

It is now well-established that the regulatory ncRNAs play a critical role in cancer, and their atypical expression leads to tumor growth and metastasis [16]. Over the years, studies have shown the aberrant expression and regulatory role of various ncRNAs in GBC. The ncRNAs associated with the development and progression of GBC are reported to be either oncogenic or tumor-suppressing in nature. The most significant of these oncogenic ncRNAs are lncRNAs, such as ANRIL, HOTAIR, LINC00152, MALAT1, and PVT1 [23,35,53,58,61]. Moreover, oncogenic circRNAs (such as circERBB2, circFOXP1, and circHIPK3), miRNAs (such as miR-663a, miR-182, miR-155, and miR-20a), and snoRNAs (such as SNORA74B) are also reported to be elevated in GBC [22,24,26,28,47,51,72,87]. Additionally, some ncRNAs in GBC also act as tumor-suppressors. For instance, lncRNA MEG3, snoRNA SNORA21, and miRNAs (such as miR-124, miR-143-3p, miR-143-5p, miR-125b, etc.) are reported in decreased levels in GBC [21,22,35,50,65,73,75]. The aberrant expression of ncRNAs, particularly miRNAs and lncRNAs, are evolving targets for a novel therapeutic approach in GBC as well as other cancers. The efficacy of the therapeutic approach is observed both alone and in combination with existing therapies. The inhibition of oncogenic miRNA function and miRNA replacement therapy are two main strategies by which miRNAs can be used as important therapeutic targets. Suppression of the function of oncogenic miRNAs is performed to inhibit their elevated levels in cancer by utilizing several approaches. One such strategic approach utilizes antisense oligonucleotides (antagomiRs, antimiRs) bind to target miRNAs and facilitate their degradation. miRNAs sponges also inhibit the function of oncogenic miRNA by blocking the interaction between miRNAs and their target. Small molecules also modulate the expression of such oncogenic miRNAs at transcription level [137]. In line with this, the suppression of the elevated levels of the miR-155 upregulated proliferation and invasion of the GBC cells. Moreover, the increased levels of miR-155 are correlated with poor prognosis. Thus, inhibiting the oncogenic miR-155 inhibited proliferation and invasion of the GBC cells [24].

In contrast, the “miRNA replacement therapy” targets the tumor-suppressive miRNAs and aims to elevate their expression in cancer. This approach introduces miRNAs as mimics or viral constructs consisting of miRNA coding genes to restore loss of function [137]. For instance, a study exploring the role of tumor suppressor miRNA, miR-30a-5p demonstrated that the introduction of miR-30a-5p mimics inhibited the migration and invasion in GBC-SD and NOZ cells [49]

Similar to miRNAs, the approaches to target lncRNAs include silencing of the lncRNA expression by antisense oligonucleotides, inhibition of molecular interaction between ncRNA and antagonistic oligonucleotide by small molecule inhibitor, and structure disruption by small molecule inhibitors, which can alter or mimic the secondary structure of lncRNA to compete for binding partners [139].The lncRNA TUG1 promoted the proliferation and metastasis of GBC cells by acting as a miRNA sponge to revoke the endogenous effect of miR-300 [71]. The inhibition of this oncogenic lncRNA will facilitate the suppression of the proliferation and metastasis of the GBC cells. Furthermore, the tumor-suppressive lncRNA MEG3 is associated with increased proliferation and decreased apoptosis of GBC cells. Inducing the overexpression of this lncRNA restores its tumor-suppressive function and decreases the proliferation of GBC cells by promoting apoptosis [53]. Thus, understanding the association of these ncRNAs in the pathogenesis of GBC may provide a future direction for improving the prognosis of this disease. Moreover, with the advancement of technology, several clinical studies have reported the presence of circulatory lncRNAs and miRNAs in the blood plasma and urine of various cancer patients [140,141]. Therefore, the presence of circulatory ncRNAs may act as a biomarker and prove beneficial in the early diagnosis of GBC.

Mounting evidence indicates that the dysregulation of ncRNAs in GBC is an indicator of the prognosis and survival of GBC patients. Research has shown that the elevated expression circRNAs are associated with the poor prognosis and survival of GBC patients [27,28]. Moreover, the upregulated expression of several lncRNAs, such as LINC00152, MALAT1, PVT1, UCA1, etc are also correlated with poor prognosis, tumor progression, lymph node invasion, and TNM stage advancement [23,35,37,61]. Additionally, the aberrant expression of miRNAs, such as miR-182, miR-155, miR-125b, miR-136, and miR-30a-5p, is correlated with lymph node metastasis and poor prognosis [24,47,48,49,50]. Moreover, ncRNAs such as HOTAIR and CCAT1 are differentially expressed in various stages of tumors, and their expression is positively correlated with the TNM stage. Thus, ncRNAs might also act as an effective diagnostic tool for the assessment of GBC progression [54,58,142].

Recently, studies have reported that some oncogenic lncRNAs regulate the hallmarks of cancer by acting as miRNA sponges as a part of the ceRNA regulatory network. For instance, the lncRNA LINC00152 and PVT1 sponge the miRNAs, miR-138 and miR-30d-5p, respectively, and thereby regulate proliferation and invasion of GBC cells [36,67]. However, studies conducted on the ceRNA networks involving different ncRNAs in GBC are limited, and therefore, a more comprehensive understanding is needed to lead to novel therapeutic targets for the treatment of GBC. Further, the ncRNAs have also been reported to target critical oncogenes, thereby regulating important hallmarks of cancer, including cell proliferation, apoptosis, EMT, invasion, migration, and metastasis [22,26,36,47,48,59,77,121,122]. Therefore, overexpressing or silencing the expression of these ncRNAs might be considered a potential therapeutic strategy that might relieve the severity of the disease and improve the overall survival of GBC patients. Additionally, a handful of studies have recently reported the involvement of ncRNAs in modulating the expression of drug resistance-related genes and regulating chemosensitivity or chemoresistance in GBC cells. It has been reported that elevated expression of miR-125b-5p, miR-218-5p, and miR-145 sensitizes GBC cells to cisplatin gemcitabine [76,80,81], whereas the overexpression of lncRNA SSTR5-AS1 facilitated chemoresistance in GBC cells to gemcitabine [69]. Thus, the chemosensitizing role of these ncRNAs can be further explored to eradicate the problem of chemoresistance in GBC. The studies mentioned above suggest that evaluation of the aberrant expression and the role of ncRNAs in patient’s tissue samples will provide a significant lead in the development of effective therapeutic strategies for GBC that will curb the problems associated with the currently available treatment modalities such as poor prognosis and survival of the patients.

## 7. Conclusions

In the current review, we discussed the emerging adverse potential of ncRNAs as a therapeutic strategy for the diagnosis and treatment of gall bladder cancer. GBC is an aggressive malignant cancer associated with late-stage diagnosis and poor prognosis and is resistant to a variety of chemotherapeutic drugs. As this cancer is generally diagnosed towards the advanced stage, both surgical and nonsurgical modes of treatment are often unsuccessful [7,9]. The lack of specific biomarkers for the diagnosis of GBC contributes significantly to the poor prognosis and patient survival rate [7]. In recent years, the role of ncRNAs has been highlighted in various diseases, including cancer. Several studies have reported the oncogenic and tumor-suppressive functions of various ncRNAs, such as lncRNAs, miRNAs, circRNAs, and snoRNAs, in GBC. The dysregulation of these ncRNAs has often been associated with the regulation of critical genes involved in the important mechanisms of cancer, such as cell proliferation, apoptosis, EMT, invasion, migration, and metastasis [21,26,47,51,53,74]. They regulate various tumor-related signaling pathways and thereby significantly contribute to the development and progression of GBC [55,61]. The current cancer therapy relies greatly on the specificity of anticancer drugs to cancer cells and their delivery and thereby possesses immense challenge. Thus, understanding the underlying mechanism of action, crosstalk between targets, as well as the functional and structural relationships of these ncRNAs in GBC, will provide novel avenues for diagnosis and targeted therapy.

## Figures and Tables

**Table 1:** Noncoding RNA expression and their effect in GBC.

ncRNAs	In Vitro/ In Vivo	Model	Target	Mechanism/Outcome	References
cirRNAs					
↑circERBB2	In vitro	GBC patient tissues, SGC-996, GBC-SD	-	↑Cell proliferation	[26]
↑circERBB2	In vivo	GBC-SD xenograft	-	↑Tumor growth	[26]
↑circFOXP1	In vitro	GBC patient tissues, NOZ, GBC-SD, EHGB-1, SGC-996, OCUG-1	↓miR-370	↑Cell proliferation, ↓apoptosis, ↓caspase-3, ↑PCNA, ↑MMP-9, ↑Akt, ↑warburg effect, ↑ECAR, ↑glycolysis, ↑PKLR	[28]
↑circFOXP1	In vivo	NOZ xenograft	↓miR-370	↑Tumor growth, ↑Ki67	[28]
↑circHIPK3	In vitro	GBC patient tissues, QBC939, GBC-SD, Mz-ChA-1	↓miR-124	↑Cell survival, ↑cell proliferation, ↓apoptosis, ↑ROCK1, ↑CDK6	[22]
**lncRNAs**					
↑AFAP1-AS1	In vitro	GBC patient tissues, GBC-SD, SGC-996, NOZ	-	Poor prognosis, ↑cell proliferation, ↑invasion, ↑migration	[52]
↑ANRIL	In vitro	GBC patient tissues, GBC-SD, QBC939	-	↓Patient survival, ↑proliferation, ↓apoptosis, ↓p53, ↑cyclin D1	[53]
↑CCAT1	In vitro	GBC patient tissues, GBC-SD, SGC-996, NOZ, EH-GB2	↓miR-218-5p	↑Bmi1 mRNA level	[54]
↑DILC	In vitro	GBC patient tissues, SGC-996, GBC-SD	-	↑Cell growth, ↑metastasis, ↑Wnt/β-catenin activation	[55]
↑DILC	In vivo	SGC-996 xenograft	-	↑Lung metastasis	[55]
↑GBCDRlnc1	In vitro	GBC-SD, NOZ	↑PGK1	Poor prognosis, ↑autophagy, ↑chemoresistance	[56]
↑HEGBC	In vitro	GBC patient tissues, SGC-996, EH-GB2, GBC-SD, NOZ	-	Poor survival, ↑cell proliferation, ↑migration, ↓apoptosis, ↑ IL-11, ↑STAT3 activation	[57]
↑HEGBC	In vivo	NOZ xenograft	-	↑Tumorigenesis, ↑metastasis	[57]
↑HOTAIR	In vitro	GBC patient tissues, GBC-SD, SGC-996, NOZ, EH-GB2	↓miR-130a	↑Cell proliferation, ↑invasion, ↑mRNA expression of c-Myc	[58]
↑HOXA-AS2	In vitro	GBC patient tissues, EHGB-1, NOZ, SGC-996, OCUG, GBC-SD	-	↑Cell proliferation, ↓apoptosis, ↑vimentin, ↑N-cadherin, ↓E-cadherin	[59]
↑H19	In vitro	GBC patient tissues, GBC-SD, EHGB-1, NOZ	↓miR-342-3p	↑Cell proliferation, ↑invasion, ↑FOXM1	[60]
↑H19	In vivo	NOZ xenograft	↓miR-342-3p	↑Tumor volume, ↑FOXM1	[60]
↑LINC00152	In vitro	GBC patient tissues, NOZ, SGC-996, EHGB-2, GBC-SD	-	↑Cell proliferation, ↑metastasis, ↓apoptosis	[61]
↑LINC00152	In vivo	GBC-SD xenograft	-	↑Tumor growth	[61]
↑LINC00152	In vitro	GBC patient tissues, GBC-SD, NOZ	↓miR-138	↓Patient survival, ↑lymph node metastasis, ↑migration, ↑EMT progression, ↑invasion, ↑HIF-1α, ↓E-cadherin, ↑vimentin	[36]
↑LINC00152	In vivo	GBC-SD xenograft	-	↑Peritoneal spreading, ↑metastasis	[36]
↑MALAT1	In vitro	GBC patient tissues, SGC-996, NOZ	-	↑Cell proliferation, ↑invasion, ↑migration	[23]
↑MALAT1	In vitro	GBC patient tissues, GBC-SD, SGC-99, NOZ	↓miR-206	↑Tumor size, ↑lymphatic metastasis, ↑proliferation, ↑invasion, ↓apoptosis, ↑ANXA2, ↑KRAS, ↑vimentin, ↓E-cadherin, ↑Twist	[62]
↑MALAT1	In vivo	NOZ xenograft	-	↑Tumor growth, ↑tumor volume, ↑ANXA2, ↑KRAS	[62]
↑MALAT1	In vitro	GBC patient tissues, GBC-SD, SGC-996, NOZ, OCUG1	-	↑Cell proliferation, ↑invasion, ↑migration, ↓ABI3BP	[63]
↑MALAT1	In vitro	GBC patient tissues, SGC-996, NOZ	↓miR-363-3p	↑Cell proliferation, ↓apoptosis, ↑MCL-1	[64]
↑MALAT1	In vivo	NOZ xenograft	↓miR-363-3p	↑Tumor volume, ↑MCL-1	[64]
↓MEG3	In vitro	GBC patient tissues, GBC-SD, QBC939	-	↑Cell proliferation, ↓apoptosis, ↓p53, ↑cyclin D1	[53]
↓MEG3	In vitro	GBC patient tissues, NOZ, GBC-SD, SGC-996, EH-GB1, OCUG-1	↓EZH2	Poor prognosis, ↑cell proliferation, ↓apoptosis, ↑invasion, ↑EMT, ↓E-cadherin, ↑vimentin, ↑N-cadherin	[65]
↑MINCR	In vitro	GBC patient tissues, NOZ	↓miR-26a-5p	↓Overall patient survival, ↑EZH2, ↑cell proliferation, ↓apoptosis, ↑invasion, ↓E-cadherin, ↑vimentin	[66]
↑PVT1	In vitro	GBC patient tissues, GBC cells	↓miR-30d-5p	↑Cell proliferation, ↑invasion	[67]
↑PVT1	In vitro	GBC patient tissues, GBC-SD, NOZ	↓miR-143	↑cell proliferation, ↑migration, ↑invasion, ↑HK2	[35]
↑PVT1	In vivo	GBC-SD xenograft	-	↑Tumor growth rate	[35]
↑ROR	In vitro	GBC patient tissues, SGC-996, GBC-SD, NOZ	-	Poor prognosis, ↑cell proliferation, ↑migration, ↑invasion, ↓E-cadherin, ↑Twist1, ↑N-cadherin	[68]
↑SSTR5-AS1	In vitro	GBC patient tissues, GBC-SD, SGC-996, NOZ	↑NONO	↑Chemoresistance, ↓apoptosis	[69]
↑SSTR5-AS1	In vivo	GBC-SD xenograft	↑NONO	↑Chemoresistance	[69]
↑SPRY4-IT1	In vitro	EH-GB1, GBC-SD, SGC-996, NOZ	-	↑Cell proliferation, ↑migration, ↑invasion, ↑E-cadherin, ↓vimentin	[70]
↑TUG1	In vitro	GBC patient tissues, EH-GB1, GBC-SD, NOZ, SGC-996	↓miR-300	↑Cell proliferation, ↑metastasis	[71]
↑UCA1	In vitro	GBC patient tissues, NOZ, GBC-SD	-	↑Cell proliferation, ↑tumor size, ↑lymph node metastasis, ↓patient survival time, ↑TNM stage, ↓E-cadherin, ↓p21, ↑EZH2 binding	[37]
↑UCA1	In vivo	GBC-SD xenograft	-	↑Tumor growth	[37]
**miRNAs**					
↑miR-663a	In vitro	GBC patient tissues, GBC-SD, NOZ	↓EMP3	Poor prognosis, ↑cell proliferation, ↑metastasis, ↑migration, ↑invasion	[72]
↓miR-143-3p	In vitro	NOZ, GBC-SD	*↑*ITGA6	↑Cell proliferation, ↑angiogenesis, ↓PLGF, Inactivation of ITAG6/PI3K/AKT pathways	[73]
↓miR-143-3p	In vivo	NOZ xenograft	-	↑Tumor growth, ↑angiogenesis	[73]
↓miR-139-5p	In vitro	GBC patient tissues, NOZ, GBC-SD	↑PKM2	Poor prognosis, ↑tumor progression	[74]
↓miR-143-5p	In vitro	GBC patient tissues, SGC-996, GBC-SD, NOZ	↑HIF-1α	↓E-cadherin, ↑vimentin, ↑Twist1, ↑VEGF	[75]
↑miR-182	In vitro	GBC-SD, EHGB1, NOZ	↓RECK	↑Cell proliferation, ↑migration, ↑invasion, ↓apoptosis, ↓caspase-3, ↓caspase-9, ↓E-cadherin, ↓Bax, ↑Bcl-2, ↑N-cadherin, ↑β-catenin	[47]
↓miR-125b	In vitro	GBC patient tissues, TYGBK-1, OCUG-1, TYGBK-8, NOZ, G-415, TGBC1TKB, TGBC2TKB, TGBC14TKB, TGBC24TKB	-	Poor prognosis, ↑cell proliferation, ↑migration	[50]
↓miR-125b-5p	In vitro	GBC patient tissues, GBC-SD, SGC-996, NOZ	↑Bcl2	Poor prognosis, ↑chemoresistance	[76]
↓miR-136	In vitro	GBC patient tissues, SGC-996, GBC-SD, Mz-ChA-1	↑MAP2K4	↑Cell proliferation, ↓apoptosis, Activation of JNK pathway	[48]
↓miR-136	In vivo	Mz-ChA-1 xenograft	↑MAP2K4	↑Tumor growth, ↓apoptosis	[48]
↓miR-324-5p	In vitro	GBC patient tissues, GBC-SD, SGC-996	↑TGFβ2	↑Invasion, ↑migration, ↑EMT	[77]
↓miR-30d-5p	In vitro	GBC patient tissues, GBC-SD, SGC-996, NOZ	↑LDHA	↑Cell proliferation, ↑invasion	[78]
↓miR-30a-5p	In vitro	GBC patient tissues, GBC-SD, SGC-996, OCUG-1, EH-GB1, NOZ	↑E2F7	↑Cell proliferation, ↑invasion, ↑migration, ↓E-cadherin, ↑vimentin	[49]
↓miR-30a-5p	In vivo	NOZ Xenograft	↑E2F7	↑Cell proliferation, ↑invasion, ↑migration	[49]
↓miR-26a	In vitro	GBC patient tissues, GBC-SD, EH-GB1, SGC-996	↑HMGA2	↑Cell proliferation	[79]
↓miR-26a	In vivo	GBC-SD xenograft	↑HMGA2	↑Cell proliferation	[79]
↓miR-218-5p	In vitro	GBC patient tissues, GBC-SD, SGC-996, NOZ	↑PRKCE	↑Chemoresistance, ↑MDR1/P-gp	[80]
↓miR-218-5p	In vivo	NOZ xenograft	↑PRKCE	↑Chemoresistance, ↑MDR1/P-gp	[80]
↓miR-145	In vitro	GBC patient tissues, GBC-SD, SGC-996	↑MRP1	↑Chemoresistance	[81]
↓miR-145	In vivo	GBC-SD xenograft	↑MRP1	↑Chemoresistance	[81]
↓miR-335	In vitro	GBC patient tissues, GBC-SD, SGC-996	↑MEF2D	↑Chemoresistance, ↑cell viability	[82]
↓miR-31	In vitro	GBC patient tissues, NOZ, NOZ/DDP, GBC-SD/DDP, GBC-SD	↑Src	↑Cell proliferation, ↑ cell viability, ↑invasion, ↓apoptosis, ↑chemoresistance	[83]
↓miR-31	In vivo	NOZ/DDP xenograft	↑Src	↑Chemoresistance	[83]
↓miR-138	In vitro	GBC patient tissues, OCUG-1, NOZ	↑Bag-1	↑Cell proliferation, ↓apoptosis	[84]
↑miR-155	In vitro	GBC patient tissues, G-415, OCUG-1, NOZ	-	↑Cell proliferation, ↑invasion	[24]
↓miR-223	In vitro	GBC patient tissues, GBC-SD, NOZ	↑STMN1	↑Cell proliferation, ↑invasion, ↑chemoresistance	[85]
↓miR-223	In vivo	NOZ xenograft	↑STMN1	↑Tumor growth	[85]
↓miR-29c-5p	In vitro	GBC-SD, NOZ	↑CPEB4	↑Invasion, ↑migration, ↑EMT	[86]
↓miR-29c-5p	In vivo	NOZ xenograft	-	↑Lung metastatic rate	[86]
↑miR-20a	In vitro	GBC patient tissues, GBC-SD	↓Smad7	↑Metastasis, ↑cell growth, ↑EMT	[87]
↑miR-20a	In vivo	GBC-SD xenograft	↓Smad7	↑Metastasis	[87]
↓miR-1	In vitro	GBC patient tissues, NOZ	-	↑Cell growth	[88]
↓miR-145	In vitro	GBC patient tissues, NOZ	-	↑Cell growth	[88]
↓miR-33a	In vitro	GBC patient tissues, GBC-SD	↑Twist1	Poor prognosis, ↑cell proliferation, ↑metastasis	[89]
↓miR-33a	In vivo	GBC-SD xenograft	-	↑Tumor growth	[89]
↓miR-1-5p	In vitro	GBC patient tissues, GBC-SD, SGC-996, HUH28, TFK-1	↑Notch2	↑Cell growth, ↑invasion, ↑migration	[90]
↓miR-1-5p	In vivo	SGC-996 xenograft	-	↑Tumor growth	[90]
↓miR-372	In vitro	GBC patient tissues, G-415, OCUG-1, SGC-996	↑CLIC1	Poor prognosis	[91]
↓miR-30b	In vitro	GBC-SD	↑NT5E	↑Cell proliferation, ↑migration, ↑invasion	[92]
↓miR-30b	In vivo	GBC-SD xenograft	-	↑Tumor growth	[92]
↓miR-340	In vitro	GBC-SD	↑NT5E	↑Cell proliferation, ↑migration, ↑invasion	[92]
↓miR-340	In vivo	GBC-SD xenograft	-	↑Tumor growth	[92]
↓miR-140-5p	In vitro	GBC patient tissues, GBC-SD	↑SEPT2	↑Cell proliferation, ↑migration, ↑invasion	[93]
↓miR-135a	In vitro	GBC patient tissues, GBC-SD, SGC-996, EH-GB1	↑VLDLR	↑Cell proliferation, ↑p38 MAPK	[94]
↓miR-135a	In vivo	GBC-SD xenograft	-	↑Cell proliferation	[94]
** snoRNA **					
↑SNORA74B	In vitro	GBC patient tissues, GBC-SD, SGC-996, NOZ, H69	-	↓Patient survival, ↑cell proliferation, ↓apoptosis, ↑Ki67, ↓PHLPP, ↓p21, ↓p27, ↑cyclin D1, ↓Bax, ↓cytosolic cyt C, ↓cleaved caspase-3, ↑Bcl-2, ↑mt cyt C, ↑Akt/mTOR	[51]
↑SNORA74B	In vivo	GBC-SD xenograft	-	↑Tumor growth, ↓apoptosis	[51]
↓SNORA21	In vitro	GBC patient tissues, GBC-SD, G415	-	↑Cell proliferation, ↓apoptosis, ↑migration, ↑invasion, ↓E-cadherin, ↑N-cadherin, ↑vimentin, ↓cleaved caspase-3, ↓Bax, ↑Bcl-2, ↓p21, ↑cyclin D1, ↑c-Myc	[21]
↓SNORA21	In vivo	GBC-SD xenograft	-	↑Tumor growth, ↑c-Myc	[21]

## Data Availability

Not applicable.

## Abbreviations

ABI3BP: ABI family member 3 binding protein, ANRIL: Antisense noncoding RNA at the INK4 locus, ANXA2: Annexin a2, Bag-1: Bcl-2-associated athanogene-1, CCAT1: Colon cancer-associated transcript-1, CDK6: rho-associated protein kinase, CLIC1: chloride intracellular channel 1, CytC: Cytochrome C, ECAR: Extracellular acidification rates, EMT: Epithelial-mesenchymal transition, EZH2: Enhancer of zeste homolog 2, GBCDRlnc1: Gallbladder cancer drug resistance-associated lncRNA1, HMGA2: High mobility group AT-hook 2, ITGA6: integrin α6, LDHA: Lactate dehydrogenase-A, MALAT1: metastasis-associated lung adenocarcinoma transcript 1, MCL-1: Myeloid cell leukaemia-1, MDR1/P-gp: Multidrug resistance 1/Permeability-glycoprotein, MEF2D: Myocyte enhancer factor 2D, MEG3: Maternally expressed gene 3, MINCR: MYC-induced long noncoding RNA, MRP1: Multidrug resistance-associated protein 1, Mt: mitochondrial, NONO: Non-POU Domain Containing, Octamer-Binding, PKLR: enzyme pyruvate kinase, liver and RBC, PRKCE: Protein kinase C epsilon, ROCK1: rho-associated protein kinase 1, UCA1: Urothelial carcinoma associated 1.
